# The Distribution, Accessibility, and Equity of Primary Care Facilities in China—A Nationwide Analysis Based on POI and High-Resolution Population Data

**DOI:** 10.3390/healthcare14142109

**Published:** 2026-07-14

**Authors:** Zhongyu He, Lu Chen, Mohammad Ghairpour

**Affiliations:** 1 School of Architecture and Urban Planning, Nanjing University, Nanjing 210093, China; hezy@nju.edu.cn (Z.H.); 502024360036@smail.nju.edu.cn (L.C.); 2 School of Architecture, Planning and Preservation, The University of Maryland, College Park, MD 20742, USA

**Keywords:** primary care, accessibility, health equity, older adults, machine learning

## Abstract

**Highlights:**

**What are the main findings?**

- Primary care facilities show significant spatial clustering and urban-rural disparity
- Horizontal equity outperforms vertical equity in serving aging populations
- Primary care accessibility is influenced by socioeconomic factors
- Machine learning identifies five city groups, with tailored strategies proposed for each

**Abstract:**

**Background**: Equitable and adequate access to primary care services is essential for reducing healthcare disparities and advancing social justice. In developing countries like China, achieving a balanced primary care provision across urban–rural divides, regions, and population groups represents a critical strategy for improving public health outcomes. **Methods**: This study integrates high-resolution population data, nationwide point of interest (POI) data, and aggregated individual survey data to analyze the spatial distribution of primary care facilities in China, evaluate their accessibility and equity, and examine the relationships among primary care accessibility, socioeconomic factors, and public health outcomes using geographic analysis and machine-learning methods. **Results**: (1) Primary care facilities in China exhibit significant spatial clustering and pronounced urban–rural disparities, with 23% of the urban population having access within walking distance; (2) while horizontal equity in primary care accessibility is relatively well-maintained for China’s aging population, vertical equity requires substantial improvement; and (3) primary care accessibility demonstrates significant but nonlinear associations with key socioeconomic indicators, including urban population size, GDP, built-up area, health insurance coverage, and public expenditure. **Conclusions**: These findings provide valuable insights for health resource allocation and urban planning policies aimed at achieving equitable primary care access.

## 1. Introduction

Primary care facilities are a fundamental component of a healthcare system. Universal health coverage is currently the aspiration of many countries worldwide, which is widely accepted as a means of achieving social justice and reducing healthcare disparities, and a strong primary healthcare system is viewed as the cornerstone of such a system [1]. During the COVID-19 pandemic when there were widespread shortages of medical resources and therapeutic strategies [2], primary care providers were on the front lines of care delivery during the outbreak and kept playing a “sentinel” role in identifying re-emerging cases after the elimination of community transmissions of the virus [3]. As a result, policymakers have realized that the adequate financing and governance of primary care services should be ensured for future health emergencies [4].

Access to primary care largely affects residents’ medical choices and thus impacts overall public health outcomes. For example, evidence shows that patients with higher spatial accessibility to primary care services made significantly more scheduled visits for asthma care [5]; individuals with good access to primary care are less likely to use the emergency department or to be hospitalized [6]. In addition, an adequately resourced primary healthcare system has been proven to substantially reduce the risks associated with cardiovascular and other diseases [7]. Therefore, achieving universal health coverage and equal access to quality healthcare has been recognized as key components of Goal 3 of the U.N. Sustainable Development Goals [8].

China launched its healthcare reform in 2009, which aimed to provide universal health coverage to all citizens, and the current healthcare system is structured around a tiered delivery model, consisting of urban tertiary hospitals, urban secondary hospitals, county-level hospitals, and primary care institutions [9]. The role of primary care in China is to provide general outpatient care as well as public health services such as vaccination and health education [10]. After more than a decade of reform, the disparities in the utilization of healthcare services across different tiers of the system remain evident. On one hand, medical workers from higher-level hospitals suffer from an excessive workload, resulting in people’s complaint about the difficulty in seeking medical treatment; on the other hand, a large number of community clinics confront the issue of insufficient patients and the underuse of medical resources. In the year 2023, primary care facility accounts for 94.39% of the country’s 1.07 million healthcare institutions; however, these facilities were responsible for only 51.73% of total outpatients’ visits, declining from 62% in 2010 [11]. This is partly due to the fact that the services provided by primary care are fragmented and widely perceived as being of inadequate quality [12]. Moreover, the unequal distribution of primary care facilities across and within various geographic regions may also contribute to the unbalanced and inefficient utilization of primary care services and, eventually, health inequities among the population. For example, in 2022, the number of community health stations per 10,000 people in the urban population was 0.77 in Zhejiang province compared to 0.18 in Yunnan province, and the life expectancy in the two regions was 80.4 and 74.4 years, respectively [13].

To address these challenges, the Chinese government has undertaken various initiatives to improve the accessibility and utilization of primary care services. In the 14th Five-year Plan for Health and the “Healthy China 2030” Planning Outline, the government pledged to “prioritize the equalization of basic health services by focusing on grassroots levels, and gradually reduce the differences in basic health services and health levels between urban and rural areas, regions, and populations to promote social fairness” [14]. To this end, much emphasis has been given to completing the primary care network, through a local health plan in which local governments determine the desired number and location of primary care facilities [15]. With an accelerating aging society in China, the burden of chronic diseases and an older population with limited mobility will significantly increase, and allocating more resources towards primary care would likely make for a more efficient healthcare system [9].

Therefore, the aim of this article is to examine the nationwide accessibility to primary care facilities among different regions and populations using finer resolution population data and point of interest (POI) data, and propose strategies to improve the equity of primary care service from a spatial perspective. This study addresses three key research questions: First, to what extent do disparities in primary care resources exist between and within different regions (e.g., provinces and municipalities), different populations, and between urban and rural areas across China? Second, what is the current state of urban primary care accessibility in China, and how is it related to various urban socioeconomic indicators? Third, what is the relationship between the equity of primary care accessibility and the self-reported health status of China’s older population?

## 2. Literature Review

### 2.1. Accessibility Measurement and Accessibility to Primary Care

Geographic accessibility to public services has emerged as an important approach to evaluating social justice in urban policy making [16]. Accessibility is influenced by factors such as the transport system, land use, travelers’ characteristics, and temporal components [17]. Several metrics have been developed to measure accessibility, including the proximity method which calculates accessibility as the travel distance (time) to the nearest destination; the cumulative opportunity approach which tallies up the number of opportunities (e.g., number of facilities) within a certain cut-off distance [16]; and the gravity-based model which is based on the seminal work of Hansen [18] and considers the trade-off between the size/quality of public facilities and travel impedance by introducing a decay function. In addition, an extended method of the gravity model, the floating catchment area method [19], has gained popularity in recent years and has been widely applied in studies on healthcare accessibility. The strength of this method, with its various incremental enhancements [20,21], is to explicitly incorporate both the capacity of the service supply and the size of the demand within a service range, giving a more balanced view of accessibility.

Employing the approaches based on either proximity, opportunity, or attraction, the physical accessibility of primary care facilities has been examined in both rural [22] and urban contexts [23], at a national [24], regional [7], or local scale [8], and generally a significant disparity of accessibility was identified in the study areas [25,26]. Among these studies, the Enhanced Two-Step Floating Catchment Area (E2SFCA) method is the dominantly employed method to evaluate accessibility; some scholars further considered integrating multi-transport mode [27] and a space–time constraint [28] into the E2SFCA method to improve the estimation accuracy of accessibility. With regard to China, most of the existing research focused on a metropolitan-level analysis, with a few carried out at the provincial level. Thus far, there is only one study [29] which quantitatively evaluated the spatial accessibility of primary care facilities across the country, and it found that 44% of communities across China had no access to a primary care facility within their 6 km catchment areas, with a more distinct disparity of accessibility observed in the north and northeastern provinces and less in southwestern and south-central provinces. However, this study did not differentiate between the types of primary care facilities or account for urban–rural variations, instead treating all facilities uniformly.

### 2.2. Equity of Healthcare Accessibility and Its Spatial and Social Correlates

Equity in the accessibility of health services is of straightforward importance in developing countries where spatial coverage problems are often obvious and acute [16]. According to the literature, primary healthcare plays a major role in the dissemination and operation of the principle of health equity [30]. The concept of “equity” needs to be established before any discussion on healthcare equity. Equity and equality are two terms often interchangeably used in research; however, they are not synonymous. The concept of equity is inherently normative—that is, value-based—while equality is not necessarily so [31]. In a widely cited paper, health inequity was defined as differences in health that are unnecessary, avoidable, unfair, and unjust [32], though not all health disparities are unfair. In the field of healthcare studies, there are typically two types of defined equity or inequity: horizontal (in)equity, which refers to the principle that people with the same healthcare needs should have similar access to healthcare services [33]; and vertical (in)equity, which accesses imbalances in healthcare accessibility among different subpopulation groups and prioritizes healthcare access for more vulnerable groups (e.g., lower income and older adults) [34]. Quantitatively, equity can be measured either by aggregate indicators such as the Gini coefficient [35], concentration curve [36], Theil index [37], Spearman’s rank correlation index [34], or individual metrics including the regression model [38], spatial autocorrelation [39], etc. By comparing the change in horizontal and vertical equities of primary care in Europe during COVID-19, Arnault et al. [40] found that horizontal equity was evident but not significantly evolved during the pandemic while vertical equity in healthcare use dramatically declined in most countries. Taking both public transit and private vehicles into consideration, Yang et al. [34] revealed greater horizontal equity in primary care facilities when using public-transit-based accessibility and higher vertical equity using private-vehicle-based accessibility in Shanghai, China.

Both spatial and social factors will impose impacts on the equity of healthcare accessibility. Land use and traffic infrastructure have been recognized as two major spatial factors affecting healthcare accessibility [41]. The imbalance allocation of healthcare resources has generated urban–rural [42] and urban core–fringe [8] inequity for accessing health services. The unavailability of alternative travel modes and traffic congestion will increase travel impedance and result in unequal access even when the travel distance is similar [43,44]; for example, Gan et al. [28] found the integration of the metro can effectively help in narrowing the accessibility gap between urban and suburban areas. Social factors—such as income, education, ethnicity, and social capital—are equally significant as spatial factors. Previous studies have identified that socioeconomically disadvantaged populations often face unequal opportunities in accessing health services [45,46]. Older adults and the disabled, who are in the need of more frequent visits to medical facilities but usually confront limited mobility [47], should obtain priority in healthcare service from the principle of both horizontal and vertical equities. The interaction between the spatial and social dimensions is also crucial to understanding equity in healthcare accessibility. For example, Pemberton et al. [48] demonstrated that minority populations in urban centers may live close to healthcare facilities, yet still face access barriers due to affordability, trust, or cultural disconnects.

The current study investigates the equity of primary care facilities in relation to spatial accessibility and its associated factors in China. Adopting a different perspective from existing research, it emphasizes the macro-level social determinants of equity and seeks to connect equity assessments with public health outcomes by integrating aggregated individual survey data with large-scale open datasets. The analysis focuses on the older population, who not only have greater primary care needs but also face significant challenges in healthcare accessibility due to limited mobility. Consequently, older adults should be prioritized for the allocation of primary care resources, regardless of whether the equity perspective is based on horizontal or vertical principles.

## 3. Materials and Methods

### 3.1. Data Collection and Pretreatment

The organization of primary healthcare in China is divided between rural and urban areas. Urban–rural classification in China is complex due to the country’s unique administrative system, rapid urbanization, and evolving criteria. We use the official government classification in this study, which is primarily based on administrative boundaries: city proper and urban districts are classified as urban, while towns and villages are classified as rural areas. However, this approach is sometimes problematic due to fuzzy boundaries and administrative division adjustment; for example, many newly designated urban districts may include rural villages. Primary care facilities in rural areas consist of village clinics and township health centers, and, in urban areas, they consist of community health centers (CHCs) and community health stations (CHSs) [12]. The principle for the distribution of primary care facilities is to include one government-run township health center in each township, and one CHC in each subdistrict (*Jiedao* in Chinese) or area of 30,000–100,000 people [15]. As of 2023, China has 37,177 CHCs and CHSs, 33,753 township health centers, and 581,964 village clinics [11]; most of these facilities are public-owned. We collected POI data of medical facilities from Gaode map API, which provided the geographic coordinates, name, category, and located county (district) of each facility. A total of 1.91 million original POI data was obtained, after data cleaning; 38,795 CHCs and CHSs, and 89,584 township health centers and village clinics were identified. The number of rural primary care facilities was significantly smaller than statistics data; the reason is that many village clinics are very small in size and are often co-located with other public facilities, such as village committees, and, therefore, their locations are not marked on map platforms. A detailed data-cleaning process can be found in Appendix A. Our final data also included 37,945 general hospitals and 140,688 private clinics.

China’s administrative divisions consist of multiple levels such as counties (districts), cities, and provinces. By 2024, mainland China has 31 provinces (including 4 provincial-level cities), 293 prefecture-level cities, and 2844 county-level administrative units (including 977 districts, 1416 counties, and 397 county-level cities). As the minimal unit of statistical data is on county (district) level, we categorized all districts and county-level cities as urban areas and all counties as rural areas. Boundary of the county level administrative units was obtained from the National Platform for Common Geospatial Information Services (https://www.tianditu.gov.cn/) and projected in ArcMap 10.8.2 (ESRI). Population data was obtained from Worldpop website (https://hub.worldpop.org). It provides the distribution of China’s population at 100 m grids, stratified by gender and age group. The population from Worldpop was adjusted according to the latest census data before imported into ArcMap. Socioeconomic data was from the City Yearbook of China 2024 (https://www.shujuku.org), which included municipal and urban districts level statistical data.

Individual health data was from China Health and Retirement Longitudinal Study (CHARLS). The CHARLS investigation, jointly carried out by Peking University and Wuhan University, was initiated in 2011 and aimed to address China’s population aging challenge and advance interdisciplinary studies on aging. Using a multistage stratified probability proportional to size sampling (PPS) procedure, the sample covered more than 17,000 respondents aged 45 years old and above from about 115 cities all over the country. Thus far, there have been 6 waves of investigations completed, from the year 2011, 2013, 2015, 2018, 2020, and 2021–2023, respectively. We used the data from the 2020 survey, which is the newest data open to the public. Questions relevant to our study included demographic information such as age, gender, marital status, educational attainment, income level, household size, residential status, and self-reported health conditions including mental health, physical health, health satisfaction, long-term care needs, and medical behavior in the past year. The sample size was 19,364, including 7765 respondents from urban areas and 11,599 respondents from rural areas. Individual data was averaged and aggregated at city level for further analysis.

### 3.2. Methodological Framework

Firstly, we used Kernel density analysis to estimate the intensity of primary care facilities across the country. Kernel density analysis is a spatial statistical technique that estimates the continuous density distribution of point or polyline features across a geographic area. And then the spatial cluster of the facilities was examined by using local indicator of spatial association (LISA); specifically, it was measured by local Moran’s I, which was defined as

(1) $$\mathrm{Moran}’s\;I_{i}=\frac{n(x_{i}-\bar{x})\sum_{j}w_{ij}(x_{j}-\bar{x})}{\sum_{i=1}^{n}{\left(x_{i}-\bar{x}\right)}^{2}}$$

where *x_i_* and *x_j_* are the numbers of primary care facilities in unit *i* and its neighboring unit *j*, $\bar{x}$ is the mean of facility numbers in all the units, and *w_ij_* is the spatial weight between unit *i* and *j*, which takes the value of 1 when *i* and *j* are adjacent and 0 otherwise. Moran’s I value varies between −1 to 1, where a positive value indicates spatial cluster while a negative value indicates dissimilar values are adjacent.

Secondly, we used Location quotient to reflect the disparity of primary care facilities between urban and rural areas, which was defined as

(2) $${\mathrm{LQ}}_{\mathrm{urban}-\mathrm{rurali}}={\mathrm{LQ}}_{\mathrm{urbani}}/{\mathrm{LQ}}_{\mathrm{rurali}}=(\frac{N_{urbani}/P_{urbani}}{N_{urban}/P_{urban}})/(\frac{N_{rurali}/P_{rurali}}{N_{rural}/P_{rural}})$$

where *LQ_urbani_* and *LQ_rurali_* are the urban and rural location quotients of city *i*, *N_urbani_* and *P_urbani_* are the number of urban primary care facilities and urban population in city *i*, and *N_urban_* and *P_urban_* are the number of urban primary care facilities and urban population of the whole country. Similarly, *N_rurali_* and *P_rurali_* are the number of rural primary care facilities and rural population in city *i*, and *N_rural_* and *P_rural_* are the number of rural primary care facilities and rural population of the whole country. This method is used to evaluate the concentration of resources with spatial attributes [49]: for example, a location quotient greater than 1 suggests that urban primary care facilities have a comparative advantage over rural areas when measured against the national average. With reference to previous studies [49,50], we divided the location quotient into 5 categories, with 0.7, 0.9, 1.1, and 1.3 as thresholds.

Thirdly, urban primary care accessibility and equity were measured using cumulative opportunity approaches. We focused on urban primary care because its location data was complete. According to the National Codes for Urban Residential Area Design (GB5018-02018) issued by the Ministry of Housing and Urban and Rural Development [51], a 15 min pedestrian-scale neighborhood should include a CHC and a 5 min neighborhood is recommended to include a CHS. Based on this, we set the service radius thresholds at 1000 m (approximately 15-min walking) for CHCs and 500 m (approximately 5 min walking) for CHSs. To estimate the population served by each facility, we employed the Mask tool in ArcMap to extract population grid cells falling within the defined service radius, and subsequently calculated the total population covered within these areas. City-level accessibility was quantified as the proportion of the urban population residing within the service areas of all primary care facilities, derived by dividing the total covered population by the total urban population. City-level equity (local equity) was assessed from the perspective of older adults; we calculated the proportion of the urban population aged 65 years and above located within the service coverage areas of all primary care facilities, namely, the accessibility of older population, and then divided it by the all-population accessibility.

The reason for using cumulative opportunity approach instead of E2SFCA is because, as mentioned earlier, primary care facilities in China are often underused; therefore, it is not necessary to incorporate the capacity of the supply side; in addition, we argued that within the service threshold we defined, closer opportunities do not differ from farther ones [52].

The Gini coefficient, a widely used statistical measure of inequity, was employed to evaluate the country-level equity (global equity) of primary care accessibility, as

(3) $$G=\frac{\sum_{i=1}^{n}\sum_{j=1}^{n}\left|x_{i}-{\;x}_{j}\right|}{2n^{2}\bar{x}}$$

where *x_i_* and *x_j_* were the accessibility scores of city *i* and *j*, $\bar{x}$ was the mean accessibility score of all the cities, and *n* was the total number of cities. The Gini coefficient is a scalar measure ranging from 0 to 1, with a lower value indicating more equitable accessibility and vice versa.

Thereafter, we used a Random Forest regression model to explore the relationship between a city’s geographic accessibility of primary care facilities and its socioeconomic characteristics. Random forest is a non-parametric machine-learning algorithm that requires minimal assumptions and is able to deal with multicollinearity between independent variables. It consists of multiple decision trees, each constructed from a randomly sampled subset of the training data. In our study, we allocated 70% of the data as a training set, while the remaining 30% was used for validating its performance and accuracy. Root mean square error (RMSE) and R-square (R^2^) were used to evaluate the model and its results. Partial Dependence Plots (PDPs) were used to illustrate the nonlinear effects of socioeconomic variables on primary care accessibility.

Lastly, we examined the relationship between the self-reported health outcomes of the older adults from CHARLS, socioeconomic development indicators and the accessibility and equity of primary care facilities, using K-means clustering method, an unsupervised machine-learning algorithm used to partition a dataset into a predefined number of clusters (k). The algorithm iteratively assigns each data point to the nearest cluster centroid, and then updates centroids based on the mean of the points assigned to each cluster. The value of K is usually decided by the elbow method.

**Figure 1:**
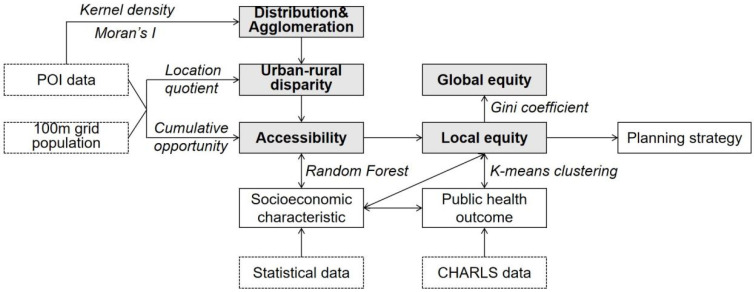
Research framework.

The methodological framework is illustrated in Figure 1.

## 4. Results

### 4.1. The Distribution of Primary Care Facilities and Its Spatial Agglomeration

**Figure 2:**
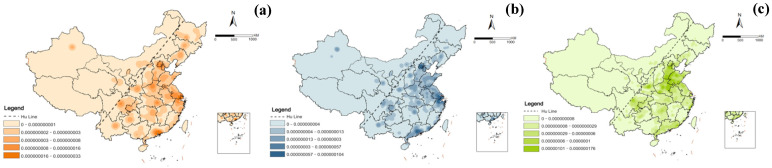
Density of (**a**) CHC, (**b**) CHS, and (**c**) township health center and village clinics.

**Figure 3:**
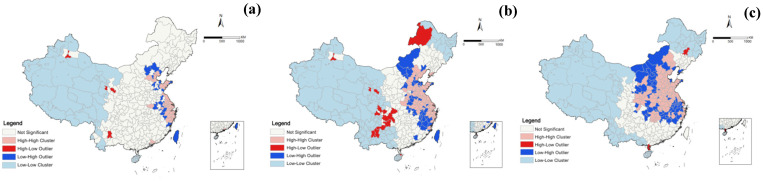
LISA of (**a**) CHC, (**b**) CHS, and (**c**) township health center and village clinics.

The densities of CHCs, CHSs, and rural primary care facilities all exhibit a similar pattern of spatial agglomeration, generally aligning with the population density, which is concentrated southeast of the Huhuanyong Line (Figure 2). Figure 3 illustrates four types of spatial agglomeration. High–High (H–H) indicates that both the spatial unit and its neighboring units have a high number of primary care facilities, while Low–Low (L–L) reflects the opposite scenario. Low–High (L–H) denotes a unit with a low number of facilities surrounded by units with higher values, and High–Low (H–L) indicates the reverse. As shown in Figure 3, both urban and rural primary care facilities exhibit H–H agglomerations along the eastern coastal areas, with L–H patterns appearing in the adjacent regions. The western regions predominantly display L–L agglomerations, while the central regions show a more scattered distribution. This pattern is further illustrated by Moran’s I values, which is 0.233 (z-score = 23.48, *p* < 0.001) for rural facilities, compared to 0.143 (z-score = 14.89, *p* < 0.001) for CHSs and 0.060 (z-score = 6.66, *p* < 0.001) for CHCs. Spatial statistical diagnostics are provided in Appendix B.

**Figure 4:**
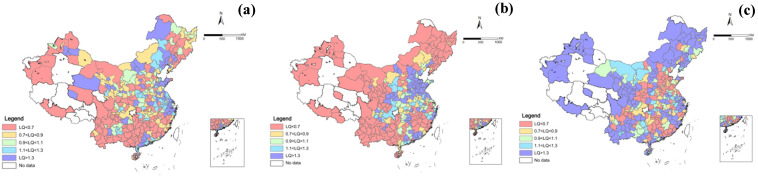
Location quotient of (**a**) urban primary care facility, (**b**) rural primary care facility, and (**c**) urban–rural disparity.

The location quotients indicate that the Yangtze River Delta, the Pearl River Delta, and the Bohai Rim Region have a higher population-adjusted density of urban primary care facilities than the national average (Figure 4a). In contrast, the central part of the country—especially Hebei, Shandong, Henan, and Shanxi provinces—as well as northern Guangdong province have a higher population-adjusted density of rural primary care facilities (Figure 4b); most western cities are less dense for both urban and rural primary care facilities compared to the national average. As a result, the urban–rural disparity of primary care facilities is quite prominent for the whole country (Figure 4c): only 0.90% of the cities have a balanced distribution of urban–rural primary care facility (0.9 < *LQ_urban-rural_* < 1.1), 46.33% of the cities have an urban-biased distribution (*LQ_urban-rural_* > 1.3), while 25.81% of the cities have a rural-biased distribution (*LQ_urban-rural_* < 0.7).

### 4.2. Self-Reported Health Status of Older Population

**Figure 5:**
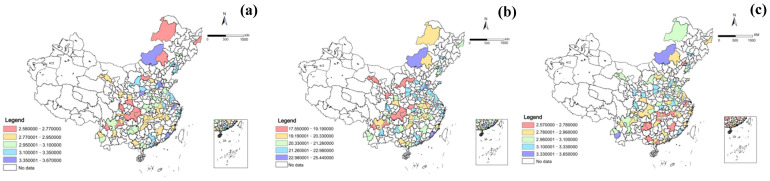
Urban resident health status: (**a**) physical health, (**b**) mental health, and (**c**) life satisfaction.

**Figure 6:**
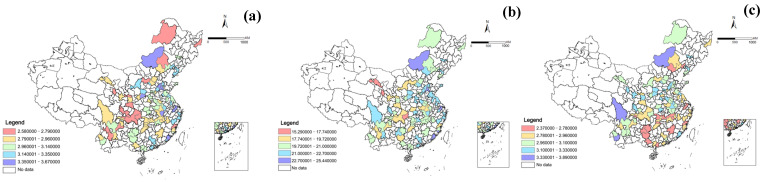
Rural resident health status: (**a**) physical health, (**b**) mental health, and (**c**) life satisfaction.

Table 1 presents the demographic and health-related characteristics of the sample from CHARLS. The average age of the sample is approximately 63 years old, with no significant difference between urban and rural areas. Gender, marital status, and the health insurance participation ratio are also similar across both urban and rural respondents. However, the urban sample has a much higher education level and household expenditure. With regard to health status, the score for chronic diseases is based on the number of 14 common chronic diseases (e.g., diabetes, hypertension, and cancer) reported by the respondent; the score for long-term care needs is evaluated by the difficulty in performing six Basic Activities of Daily Living (BADL) including eating, bathing, dressing, toileting, continence, and transferring. Overall, urban residents report a slightly better self-rated physical health, as well as a notably better mental health status and a lower score for long-term care needs. However, urban residents’ life satisfaction is slightly lower than their rural counterparts. There is no difference in the chronic diseases scores between the two groups. The self-rated physical health and mental health status are negatively related for both urban and rural samples. Residents from coastal cities tend to have a better physical health but lower healthy mental status than inner-city residents, and residents living in southern China have a lower life satisfaction than residents living in the north (Figure 5 and Figure 6).

### 4.3. Measuring Accessibility and Equity of Urban Primary Care Facilities

**Figure 7:**
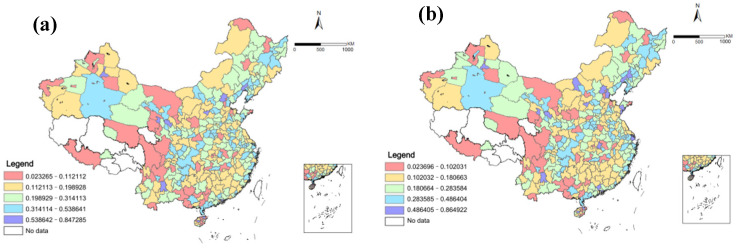
Accessibility of (**a**) all population, and (**b**) older population.

**Figure 8:**
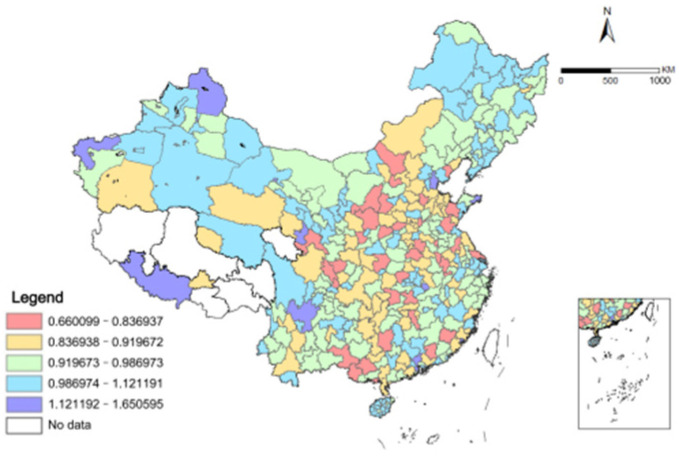
Local equity of urban primary care facility.

**Figure 9:**
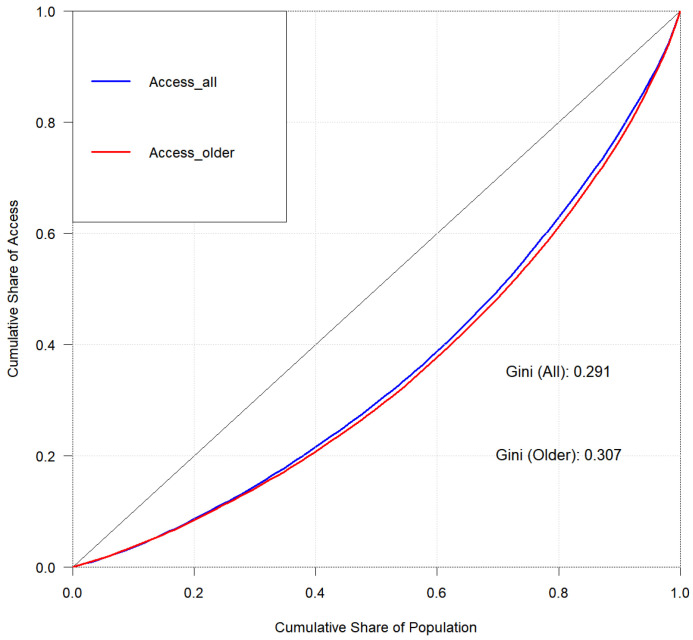
Lorenz curve of urban primary care accessibility.

Using the previously described method, we calculated the accessibility of urban primary care facilities for the general population and older population, which yielded very similar results (Table 2). On average, approximately 23% of the general population and 22% of the older population in urban areas are covered within 1000 m of a CHC or 500 m of a CHS, respectively. The most accessible city is Urumqi and the least accessible city is Ganzi Tibetan Autonomous Prefecture. In terms of local equity, which was measured as the ratio of the general population accessibility to the older population accessibility, the most inequitable city is Jincheng, while the most equitable city is Zhongshan. Spatially, the accessibility and equity distribution of urban primary care facilities is relatively dispersed (Figure 7 and Figure 8). High accessibility does not necessarily indicate a high equity level. Notably, although the northeastern and western regions of the country have a lower density of primary care facilities, their equity scores are above the average level of the country. Figure 9 presents the Lorenz curves for global equity; Gini coefficients of 0.291 for the general population and 0.307 for the older population indicate a moderate level of inequity, with accessibility for the older population exhibiting a slightly higher degree of inequity.

### 4.4. Random Forest Regression

To examine the relationship between the accessibility of urban primary care facilities and macro-level socioeconomic characteristics, we selected 11 indicators representing three key dimensions: urban size, economic development, and healthcare service capacity (Table 3). Urban population was calculated by summing the populations of all urban districts within each city. Strictly speaking, this is not entirely equivalent to the total urban population, as some urban residents may live in counties, and a small proportion of rural residents may reside in urban districts. However, since our statistical data is only available at the county (district) level, this approach provides the best possible alignment with the available socioeconomic data. There are different types of social security coverage and health insurance in China; here, we consider employee pension insurance and employee medical insurance, a more privileged type of insurance. The socioeconomic data showed great varieties across different cities, especially for urban gross domestic product (GDP) and annual public expenditure.

**Figure 10:**
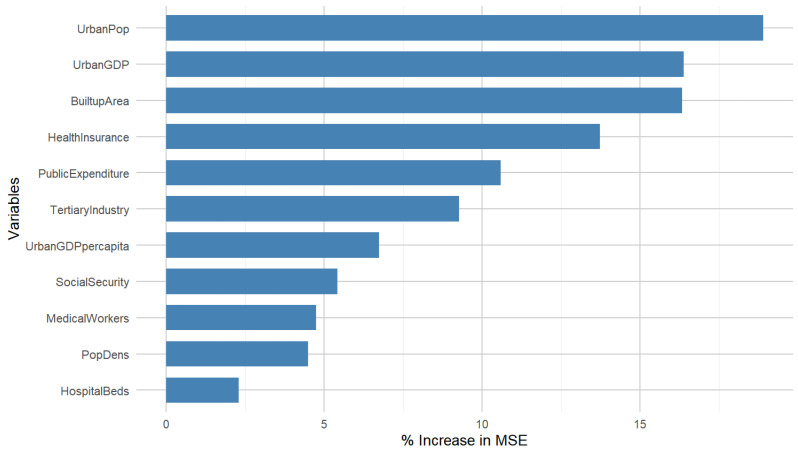
Importance of variables.

**Figure 11:**
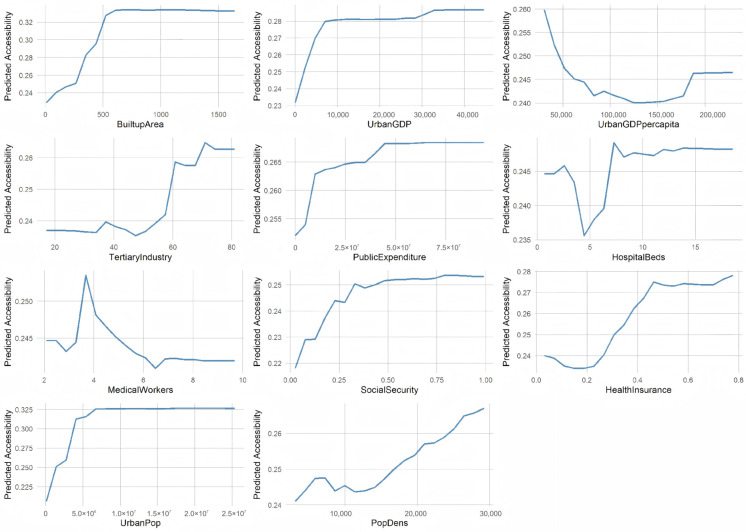
Partial dependence plot of the independent variables.

A random forest regression model was implemented in R 4.3.0 (https://www.r-project.org/) to analyze these relationships. After excluding samples with incomplete data, a total of 289 cities were retained for modeling. The optimal mtry was identified via a grid search ranging from 2 to 10 (in increments of 2), with the objective of minimizing the root mean square error (RMSE) obtained from 10-fold cross-validation. The number of trees was chosen by monitoring the convergence of the out-of-bag error as a function of the tree count. The hyperparameters of the final model are summarized in Table 4. The R-square and root mean square error were 0.352 and 0.117, respectively. Figure 10 and Figure 11 illustrate the ranking of importance of the 11 independent variables and their partial dependence plots (PDPs). The urban population size is mostly related with primary care accessibility, followed by urban GDP; the number of hospital beds is the least related factor. Generally, the urban size and economic development level have more impacts on accessibility than healthcare service capacity. The health insurance participation rate is the only factor reflecting healthcare service that has a major impact. All independent variables showed a nonlinear relationship with accessibility, and a majority of the relation was positive. The urban GDP per capita exhibited a negative correlation: the higher the value was, the less accessible the primary care facility was, contrary to common expectations. However, the Pearson correlation of the two variables showed a positive coefficient of 0.401 (*p* < 0.01). This apparent paradox was due to the high multicollinearity between GDP per capita and other independent variables, which might exert higher impacts on accessibility (Table 5). For example, when the total GDP is held constant, a higher GDP per capita indicates a smaller population size, which may reduce the accessibility more substantially. In addition, two variables (i.e., HospitalBeds and MedicalWorkers) presented a non-monotonic correlation.

### 4.5. K-Means Clustering

**Figure 12:**
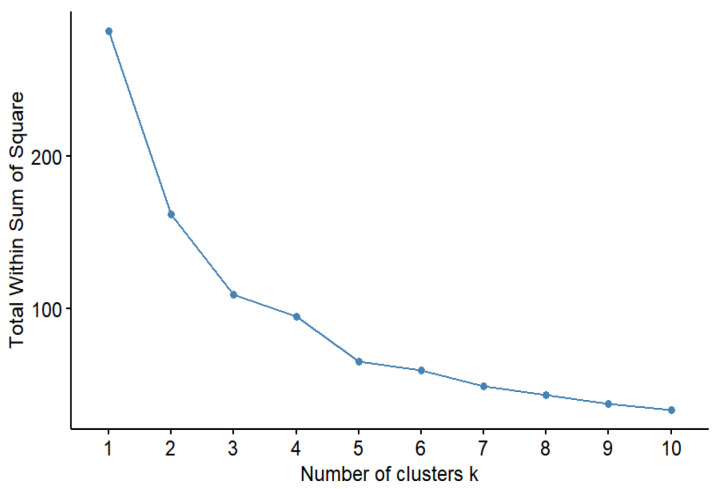
Result of the elbow method.

Before implementing the K-means clustering in R, we carried out a principal component analysis (PCA) to reduce the dimensionality of the variables, and three principal components, health status, access and equity, and socioeconomic features, were generated and then standardized. The elbow method (Figure 12) and silhouette coefficient (=0.3339) indicated a preferable K value of 5.

**Figure 13:**
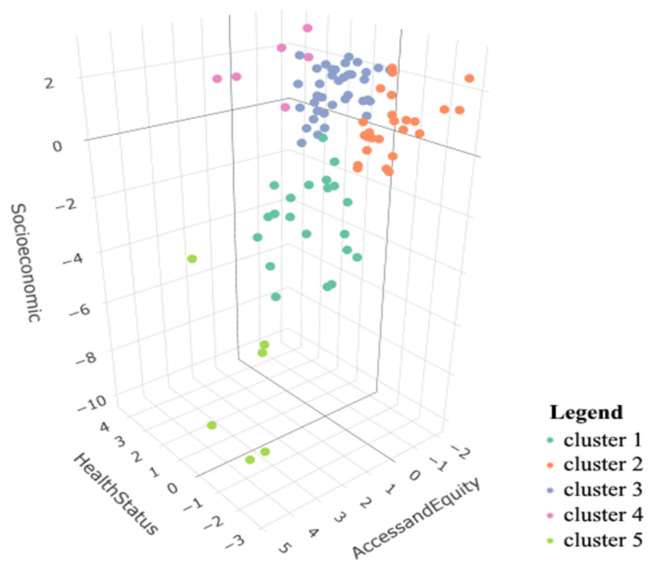
Visualization of the city cluster.

Based on the results of the K-means cluster analysis, the sampled cities can be classified into the five clusters: Cluster 1: These cities exhibit high levels of socioeconomic development, strong accessibility and equity in primary healthcare services, and favorable self-reported health outcomes. Cluster 2: These cities maintain moderate socioeconomic development and limited primary healthcare coverage, yet achieve relatively high levels of self-reported health—demonstrating a capacity to deliver better health outcomes with fewer resources. Cluster 3: This category comprises cities where socioeconomic conditions, healthcare accessibility, and self-reported health are generally aligned but uniformly low, indicating a need for comprehensive improvement across all dimensions. Cluster 4: Cities in this group are characterized by low socioeconomic development, poor accessibility and equity in healthcare services, and the lowest levels of self-reported health, highlighting the urgent need for targeted interventions and resource allocation. Cluster 5: Cities in this group demonstrate advanced socioeconomic and primary healthcare development; however, the self-reported health levels remain only moderately high, suggesting a lag in perceived health benefits (Figure 13).

## 5. Discussion

### 5.1. Regional Disparity of the Distribution of Primary Care Facilities

The density of primary care facilities in China aligns with the population distribution and exhibits a clear pattern of spatial agglomeration. Rural primary care facilities display a more pronounced and widespread agglomeration, particularly in the northern and central regions of the country. In contrast, urban primary care facilities are clustered more tightly around major metropolitan areas, forming smaller but denser groupings. Notably, although the western and northeastern regions of China have a lower number and density of primary care facilities, the facilities are more concentrated in urban areas, which generated a strong urban–rural disparity. Existing studies have reported mixed results regarding the urban–rural disparity of healthcare access: for example, in England and Germany, urban areas were found to have a significantly higher accessibility to primary care than rural areas [24,25], while, in Australia, the spatial accessibility to all public hospitals in remote and very remote areas was not lower (and may be even higher) than that in major cities [42]. In our study, we did observe a significant disparity; however, it was not a straightforward case of an urban advantage. In approximately one-fourth of the cities, rural areas actually exhibited a considerable comparative advantage in facility density compared to urban areas. This finding stems from our use of a relative measurement approach—namely, the location quotient—whereas the aforementioned studies relied on absolute metrics. We argue that, from the perspective of public service efficiency, it is questionable to expect rural areas to maintain the same standard of medical services as urban areas. In addition, in the context of China where there is a dual primary care system for urban and rural areas, a relative comparison is more appropriate and meaningful.

The pattern observed in China is also shared by other countries with significant regional variation, such as India, Brazil, or the United States, all of which show a coastal/developed core agglomeration, inland/remote deprivation, and urban–rural disparity. However, China differs from India in its substantially stronger public primary care infrastructure [53] and from the United States in its near-universal insurance coverage [54]. Among the comparator countries, Brazil’s Family Health Strategy represents the closest analogue to China’s recent efforts to strengthen primary care gatekeeping, although Brazil has achieved a stronger continuity of care and community-based service integration [55]. Against this unbalanced distribution of primary care resource, artificial intelligence (AI) is emerging as a cross-national leveling mechanism capable of mitigating structural spatial disadvantages in grassroots healthcare supply and access [56]. For instance, AI-powered telehealth services have expanded rapidly across China’s primary care system, with rural and county-level residents constituting the majority of users of mainstream digital medical platforms, alleviating shortages of general practitioners in remote regions [57].

### 5.2. Social Correlates of Accessibility to Primary Care

With regard to primary care accessibility, our study shows, on average, about 23% of the total urban population in China have access to CHCs within a 1 km service radius or CHSs within a 500 m radius. In a study by Peng et al. [29], it was found that about 22% of communities across China, representing approximately 31.6% of the overall population, had access to primary care service within their 1.5 km catchment areas. Both studies highlight a persistent gap between governmental targets and actual service coverage, indicating significant room for improving the accessibility of primary care facilities. When we further examined the local equity from the perspective of older adults, which was defined by the ratio of accessibility by the general population to the older population, we found most regions had a matched primary care coverage for the older and general populations, suggesting good local equity from the horizontal perspective. Regions with lower local equity include the Shanxi, Shaanxi, Hebei, Shandong, Anhui, Guangxi, and north Jiangsu provinces. Notably, the northeastern and western region which have a lower density of primary care facilities exhibit a high level of local equity. However, from the standpoint of vertical equity, additional primary care resources should be directed to areas with higher concentrations of older adults within a city, to improve the accessibility of the more vulnerable and mobility-challenging population group.

Regarding global equity, our study reveals a Gini coefficient of 0.29 and 0.31 for the general population and the older population, respectively. A comparable study found that the Gini coefficient of primary care institutions by population size in China was 0.26–0.27 between the year 2011–2014 [58]. This indicates the equity of primary care accessibility has barely seen improvements despite over a decade of healthcare reform. Other studies showed that, in Japan and Britain, the Gini coefficients of primary care physicians against the population were 0.175 and 0.083 [59]; although these figures were based on the number of physicians rather than facilities and, therefore, not directly comparable, they still highlight a significant disparity between China and developed countries.

The random forest model provided the ranking of macro-socioeconomic characteristics based on their correlation with primary care accessibility, and, more importantly, it revealed the nonlinear relationship between socioeconomic conditions and accessibility. Consistent with previous studies [7], we found that urban size (e.g., area and population density) and economic development (e.g., GDP) had a close correlation with primary care accessibility. The difference is that their study found that area had a negative impact while our result showed a positive one, and this is because, in Wang’s study, they used the total administrative area and we used the urban built-up area. Cities with a larger administrative area in China typically have a smaller built-up area and are usually less developed in terms of economy. The influence of the built-up area, urban GDP, public expenditure, and urban population performed similarly: initial increases in these variables were strongly associated with improved accessibility, but, beyond a certain threshold, further increases had little to no additional impact. On the other hand, population density had a constant impact, and the denser the population of a city is, the better accessibility its residents will have. This result further underscores the efficiency of high-density urban development in enhancing public service accessibility. A third type of nonlinear effect involved variables like the population-adjusted number of hospital beds and medical workers, which showed a “V” or inverted “V” shape. For example, a city with a low number of medical workers likely lacks sufficient investment in healthcare services, while a city with a very high number may have a sparse population or may prioritize higher-level facilities, such as hospitals, over the development of primary care services.

### 5.3. Primary Care, Socioeconomic Conditions, and Older Adults’ Health

The self-reported health outcome from CHARLS indicates that the surveyed older adults in China have a moderate level of physical health and life satisfaction, and the majority of them do not have long-term care needs with limited chronic diseases. There was a small gap for the self-reported health between urban and rural areas. And the samples’ physical and mental health status show a negative relationship. We explored the interaction between the urban older adults’ health and the social and spatial dimensions of urban primary care, and found that health outcome had no significant correlation with either primary care accessibility/equity or socioeconomic conditions. This might be due to the heterogeneity of Chinese cities regarding the complicated interplay between the three factors; therefore, we tried to break all cities into smaller groups.

K-means clustering identified five distinct city clusters, and we named these clusters, based on the relationship among primary care accessibility, health status, and socioeconomic features in each cluster, as follows: (1) the Coordinated Advantage Type (cluster 1), where socioeconomic development, primary care accessibility, and public health have progressed in a balanced and integrated manner—this consists of 21 cities, including 11 province capital cities and 8 s-tier cities such as Suzhou, Dalian, Ningbo, etc.; (2) the Senior-friendly Type (cluster 2), characterized by livable conditions for older adults and a high level of perceived health status—this group contains 25 cities, most of them which third-tier cities with a moderate size, and the majority of them (15 out of 25) are located in the more developed coastal provinces, such as Weihai, Yangzhou, Jiaxing, Huzhou, etc., with the rest of the cities being mostly adjacent to provincial capital cities in central China; (3) the Coordinated Improvement Type (cluster 3), where public health outcomes can be further enhanced through improvements in economic development and healthcare infrastructure—this comprises 37 cities (making this the largest cluster), which are predominantly third-tier cities in the northeast, central, and western regions, including Jilin, Ganzhou, Chifeng, Jixi, etc.; (4) the Urgent Improvement Type (cluster 4)—these six cities face significant challenges in healthcare services and low levels of perceived health, and they include Shangrao, Jiujiang, Zhangye, Yulin, Dingxi, and Xiangyang, all located in central and western China.; and (5) the Health Lagging Type (cluster 5), where the health status of the residents lags behind cities’ socioeconomic and healthcare development—there are 6 cities in this group, including the 4 first-tier cities (i.e., Beijing, Shanghai, Guangzhou, and Shenzhen), a provincial-level city (Tianjin), and a province capital city (Lanzhou).

These five groups demonstrate that, generally, a higher administrative status and larger cities benefit more from the allocation of primary care resources, resulting in better health outcomes for their residents. However, some medium-size or smaller cities which are located in the developed coastal regions or close to higher-level cities such as provincial capitals also manage to provide residents with a good quality of life and relatively high perceived health, even if their primary care systems are less developed. On the other hand, for the largest and most developed cities, while the life expectancy is high, perceived health does not always align with the level of healthcare services available—possibly due to the high stress and fast-paced lifestyle in these urban centers.

### 5.4. Strengths and Limitations

By integrating high-resolution population data at a 100 m grid scale with nearly 140,000 POI data, this study represents one of the few national-scale analysis of the spatial distribution and accessibility of primary care facilities in China using quantitative methods. Furthermore, it is the first to investigate the interplay between primary care accessibility, socioeconomic factors, and health outcomes by incorporating data from a nationwide survey targeting older adults in China. Methodologically, we employ a random forest model to identify the macro-level socioeconomic correlates of primary care accessibility and examine their nonlinear effects.

This study has a couple of limitations: (1) The method used to access accessibility was simplified: we only considered the Euclidean distance and walking mode when evaluating the coverage of primary care facilities. A future study may use road network data to more precisely calculate the travel distance and incorporate time constraints and multimodal transportation options to better reflect spatial accessibility. (2) In our analysis of equity, we prioritized older adults due to their heightened medical needs and mobility constraints; the equity analysis could be expanded to other vulnerable groups—such as children, and individuals with chronic diseases, especially low-income populations—pending data availability. (3) Spatial accessibility does not always equate to actual accessibility: indicators of primary care facility quality, such as the number of doctors, the standard of equipment, and the range of services provided can be taken into consideration where data is available. (4) The cross-sectional research design might limit the generalizability of the conclusions; a more robust causal inference can be obtained by employing longitudinal data. For example, a future study can compile multi-year datasets of primary care facility POIs and examine how the change in the facilities is affected by social and economic development and how it is related with the change in people’s health outcome.

## 6. Conclusions

The distribution of primary care facilities in China presents a clear spatial agglomeration, with a higher density in the coastal region and lower density in the western region. The urban–rural disparity is evident, as measured by the Location quotient: in 46.33% of the cities, the population-adjusted urban–rural ratio of primary care facilities is significantly higher than the national average, and 25.81% of the cities display the opposite trend. Regarding primary care accessibility, our analysis is based on the locations of the whole country’s 38,795 CHCs and CHSs, and we found that about 23% of the total urban population in China have access to CHCs within a 1 km distance from home or CHSs within a 500 m distance. Primary care accessibility for the general population versus the older population does not show a significant difference, and the Gini coefficients for the two population groups are 0.29 and 0.31, respectively. Primary care accessibility is closely and positively associated with macro-socioeconomic characteristics. Among these, urban population, urban GDP, built-up area, health insurance participation rate, and public expenditure exhibit the strongest influence, and the relationship is nonlinear—characterized by a rapid initial improvement in accessibility as these factors increase, followed by a plateau once a certain threshold is reached. We further found that Chinese cities exhibit a substantial heterogeneity regarding the interaction between residents’ health outcome, primary care accessibility, and socioeconomic development, leading to the identification of five distinct city clusters.

These findings yield policy implications for urban-planning practitioners: at the national level, policies to reduce the regional gap of primary care accessibility should be initiated, and the enhancement in rural primary care service in western and northeastern China should be prioritized, as these regions face not only a lower facility density but also pronounced urban–rural inequities. In remote rural areas where establishing primary healthcare institutions is unfeasible, promoting telemedicine and similar modalities should be encouraged to enhance healthcare accessibility. At the local level, primary care coverage remains inadequate relative to government targets and healthcare reform objectives, especially in moderate-sized cities in the northeast, central, and western regions of the country. Therefore, primary care service should be strengthened by the planning of a “15 min community-life circle”. Meanwhile, additional primary care resources should be directed to areas with a larger older population to promote the vertical equity of primary care in China. To date, most primary care institutions are public in China; for cities with a constrained public fiscal capacity, the government should attract private primary care providers by offering financial incentives such as a tax reduction. Finally, urban planning should emphasize high-density development—particularly in underserved newly developed districts—to enhance the efficiency and spatial accessibility of primary care services.

## Figures and Tables

**Table 1:** Demographic feature and self-reported health status of the sample older adults.

Variable		Percentage (%) or Mean	
		Urban	Rural
Age (years old)		63.27	63.36
Gender	0: Male	53.58%	52.80%
	1: Female	46.42%	47.20%
Education	1: Elementary school and below	30.16%	51.69%
	2: Junior middle school	20.94%	22.60%
	3: High school and above	48.90%	25.71%
Marital status	0: Single or widowed	15.92%	16.17%
	1: Married	84.08%	83.83%
Household expenditure per capita (yuan/year)		23,775.54	16,467.74
Health insurance	0: No insurance	4.25%	5.18%
	1: Have an insurance	95.75%	94.82%
Self-rated physical health (1: very unhealthy to 5: very healthy)		3.13	2.99
Life satisfaction (1: very unsatisfying to 5: very satisfying)		2.92	2.99
Mental health (0: very healthy to 30: very unhealthy)		7.65	9.38
Score of chronic diseases		2.28	2.27
Score of long-term care needs (0: no need to 6: very high need)		0.40	0.54

**Table 2:** Descriptions of the accessibility and local equity.

Variable	Maximum	Minimum	Mean	Standard Deviation
Accessibility_all	0.8473	0.0233	0.2300	0.1348
Accessibility_older	0.8649	0.0237	0.2198	0.1355
Local equity	1.3476	0.6601	0.9547	0.0963

**Table 3:** Descriptions of independent variable in random forest.

Independent Variable		Definition	Mean	Std.
Urban size	UrbanPop	Urban population (10,000 people)	214.99	336.29
	BuiltupArea	Size of buildup area in urban districts (km^2^)	179.10	238.94
	UrbanPopDens	Urban population density (people/km^2^)	12,234.26	5170.86
Economic development level	UrbanGDP	Urban gross domestic product (100 million yuan)	2493.31	5282.30
	UrbanGDPpercapita	Urban gross domestic product per capita (yuan)	86,867.05	41,556.85
	TertiaryIndustry	Proportion of tertiary industry in urban GDP	0.53	0.10
	PublicExpenditure	Annual public expenditure for urban area (1,000,000 yuan)	32,904.78	83,827.93
Healthcare service capacity	MedicalWorkers	Number of licensed doctors per 1000 urban population	4.07	1.11
	HospitalBeds	Number of hospital beds per 10,000 urban population	7.84	2.52
	HealthInsurance	Urban health insurance participation rate	0.29	0.17
	SocialSecurity	Urban social security coverage rate	0.37	0.18

**Table 4:** Hyperparameter settings of the random forest model.

Mtry	Ntree	Nodesize	Maxnodes	Seed
2	500	5	Null	123

**Table 5:** Pearson correlation between GDP per capita and other socioeconomic variables.

	Urban Pop	Urban GDP	Builtup Area	Health Insuran	Public Expen	Tertiary Indust	Social Security	MedicalWorker	Pop Density	Hospital Bed
GDP Per capita	0.41 *	0.52 *	0.45 *	0.50 *	0.42 *	−0.16 *	0.46 *	0.01	−0.10	−0.22 *

* *p*-value < 0.01.

## Data Availability

Data is available from the corresponding author upon reasonable request.

## Abbreviations

The following abbreviations are used in this manuscript:

CHCs	Community health centers
CHSs	Community health stations
E2SFCA	Enhanced Two-Step Floating Catchment Area
POIs	Points of interest
CHARLS	China Health and Retirement Longitudinal Study
LISA	Local indicator of spatial association

## Appendix B. Global Moran’s I Significance Tests

Global Moran’s I of CHS (left), CHC (middle), and rural primary care facilities (right).
